# A Novel Metallo‐β‐Lactamase AMM‐1 From *Alteromonas mangrovi* Reveals a Cryptic Environmental Reservoir of Carbapenem Resistance

**DOI:** 10.1111/1751-7915.70191

**Published:** 2025-07-08

**Authors:** Xuan Wu, Xinjing Han, Lu Zhu, Ningning Pi, Yi Li, Rong Xiang

**Affiliations:** ^1^ Key Laboratory of Environmental Chemistry and Ecotoxicology of Organic Pollutants of Chongqing Chongqing Ecological and Environment Monitoring Center Chongqing China; ^2^ College of Environment and Ecology (CEE) Chongqing University Chongqing China; ^3^ Department of Gastroenterology The Second Affiliated Hospital of Chongqing Medical University Chongqing China; ^4^ Key Laboratory of Clinical Laboratory Diagnostics (Ministry of Education), College of Laboratory Medicine Chongqing Medical University Chongqing China; ^5^ Western (Chongqing) Institute for Digital‐Intelligent Medicine, Chongqing National Biomedicine Industry Park Chongqing China; ^6^ Precision Medicine Center The Second Affiliated Hospital of Chongqing Medical University Chongqing China

**Keywords:** AMM‐1, carbapenemase, environmental reservoirs, metallo‐β‐lactamases

## Abstract

Carbapenem resistance driven by metallo‐β‐lactamases (MBLs) poses a formidable global challenge as these enzymes can degrade a wide range of β‐lactam antibiotics, including last‐line carbapenems. Despite extensive documentation of MBL‐producing pathogens, their evolutionary origins and ecological reservoirs are still poorly understood. Here, we report the discovery and in‐depth characterisation of AMM‐1, a previously unrecognised B1.2 MBL identified within a metagenome‐assembled genome of *Alteromonas mangrovi* obtained from the Yangtze River Estuary. Comparative sequence analyses and phylogenetics reveal that AMM‐1 clusters closely with clinically significant MBLs, underscoring its potential impact to human health. Structural modelling confirms the presence of a conserved di‐zinc binding site critical for β‐lactam hydrolysis, while heterologous expression in *Escherichia coli* ( *E. coli* ) demonstrates a marked increase in resistance against multiple β‐lactam classes, including carbapenems. Phylogenetic depth analysis and ancestral reconstruction delineate AMM‐1's distinct evolutionary path, placing it deeper than IMP‐1 and SPM‐1 but shallower than NDM‐1. Flexibility simulations reveal unique active‐site loop dynamics (L3 and L10), with reduced mobility in key regions that shape substrate binding stability and spectrum. Notably, AMM‐1 is stably located on the host chromosome without flanking mobile genetic elements, suggesting that it may have persisted as a vertically inherited trait rather than a recently acquired component of a mobile resistome. These findings highlight the capacity of environmental microbes to serve as long‐standing, cryptic reservoirs of potent resistance determinants, emphasising the need for integrated environmental surveillance and preemptive stewardship strategies. By unveiling the molecular and functional properties of AMM‐1, this work provides critical insights into how resistance elements can reside, evolve and potentially mobilise within natural habitats, ultimately informing efforts to predict and mitigate the future emergence of carbapenem‐resistant bacterial pathogens.

## Graphical abstract

We identified and characterised AMM‐1, a novel metallo‐β‐lactamase from Alteromonas mangrovi in the Yangtze River Estuary. AMM‐1 clusters with clinically relevant MBLs. Structural modelling reveals a conserved di‐zinc active site essential for β‐lactam hydrolysis; AMM‐1 shows distinct active‐site flexibility compared with representative MBLs, potentially affecting substrate recognition and resistance evolution.

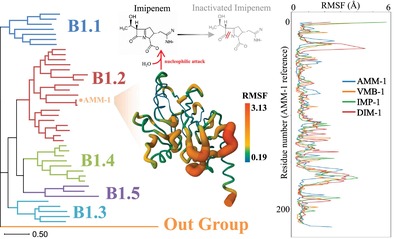

## 1 Introduction

The proliferation of antimicrobial resistance (AMR) genes constitutes a critical global health crisis, eroding the efficacy of antibiotics once considered last‐resort therapies. Recent modelling estimates that bacterial AMR was associated with 4.95 million deaths worldwide in 2019, making it one of the leading infectious killers (Murray et al. 2022). Brüssow (2024) further warns that, without coordinated global action, AMR‐related mortality could surpass that of cancer by mid‐century, underscoring the urgency of tracking resistance determinants across clinical and environmental compartments. Among the many forms of resistance that have emerged, β‐lactamases stand out due to their capacity to hydrolyse a wide range of β‐lactam antibiotics, thereby threatening the cornerstone of modern infectious disease treatment (Khan et al. 2017). Carbapenems, in particular, have long been relied upon as a final line of defence against multidrug‐resistant bacterial pathogens. However, their utility is now jeopardised by the expanding prevalence of carbapenem‐hydrolysing metallo‐β‐lactamases (MBLs) (Bonomo et al. 2018). These MBLs, which rely on zinc ions for catalytic activity, can degrade a broad spectrum of β‐lactams, including carbapenems, greatly diminishing our therapeutic arsenal (Tooke et al. 2019).

While clinically relevant MBLs, such as NDM, IMP and VIM variants, are well documented in healthcare‐associated pathogens, mounting evidence suggests that these resistance determinants often originate in non‐clinical environments (Marathe et al. 2017; Hendriksen, et al. 2019; Parnanen et al. 2019). Natural habitats, including aquatic ecosystems, soils and animal microbiomes, harbour a rich diversity of antibiotic resistance genes (ARGs), many of which predate clinical antibiotic usage (Finley et al. 2013; Berendonk et al. 2015; Larsson and Flach 2022). Environmental compartments, especially those exposed to anthropogenic influences such as wastewater discharge, agricultural runoff and industrial pollutants, serve as breeding grounds for the selection and enrichment of resistant strains (Iwu et al. 2020; Serwecińska 2020; Larsson and Flach 2022). Under these conditions, mobile genetic elements (MGEs) such as plasmids, integrons and transposons can facilitate horizontal gene transfer (HGT), enabling AMR genes to move across species and even cross significant phylogenetic boundaries (Ebmeyer et al. 2021; Forster et al. 2022; Ghaly and Gillings 2022).

Estuarine and coastal regions represent particularly dynamic interfaces where freshwater and marine microbial communities intersect, often under the substantial influence of human activities (Guo et al. 2019). These transitional zones can accumulate various pollutants—including residual antibiotics and heavy metals—that create selective pressures fostering the persistence and spread of resistance determinants (Chen et al. 2019). Understanding how resistance genes evolve and persist in such environments is crucial to anticipating their eventual mobilisation into clinically significant pathogens. Indeed, recent studies have shown that genes conferring carbapenem resistance, once confined to environmental bacteria, have successfully transitioned into Enterobacteriaceae and other human‐associated pathogens, fueling hospital outbreaks and community‐acquired infections (Khan et al. 2017; Marathe et al. 2017; Hendriksen, et al. 2019; Parnanen et al. 2019).

Despite growing recognition of the environment's critical role in shaping the global resistome, the evolutionary trajectories and molecular contexts that enable environmental AMR genes to infiltrate clinical ecosystems remain incompletely understood. Characterising novel MBLs directly from environmental sources can provide valuable insights into their structural features, potential mobility and functional capacities. Such knowledge informs not only the mechanistic underpinnings of resistance but also helps refine surveillance strategies, ultimately contributing to more proactive interventions aimed at preserving the therapeutic efficacy of our existing antibiotics (Huijbers et al. 2019).

In this study, we investigated microbial communities from the Yangtze River Estuary, a key ecological and economic corridor in China known for significant anthropogenic inputs. Through metagenomic assembly, we discovered a novel B1.2 subclass MBL gene, designated AMM‐1, located in an *Alteromonas mangrovi* ( *A. mangrovi* ) genome. We conducted a comprehensive analysis encompassing phylogenetics, comparative genomics, in silico structural modelling and functional assays in a heterologous host. Our results reveal close evolutionary and structural relationships between AMM‐1 and clinically significant MBLs, despite the gene's current stable chromosomal integration and lack of flanking MGEs. These findings underscore the potential for environmental reservoirs to harbour clinically relevant resistance determinants and highlight the importance of integrating environmental and clinical perspectives in combating the escalating global AMR crisis.

## 2 Materials and Methods

### 2.1 Sample Collection and Metagenomic Sequencing

Water samples were collected from the Yangtze River Estuary in Shanghai, China, to investigate the microbial communities present in this aquatic environment. Sampling was conducted at designated sites using sterilised 10‐L polyethylene containers. The samples were immediately placed on ice and transported to the laboratory within 6 h of collection. Microbial biomass was concentrated by filtering the water samples through 0.22 μm pore‐size membrane filters (Millipore, USA). Total genomic DNA was extracted from the filters using the DNeasy PowerSoil Kit (Qiagen, Germany) following the manufacturer's protocol. The extracted DNA was quantified using a Qubit 2.0 Fluorometer (Thermo Fisher Scientific, USA) and assessed for purity with a NanoDrop 2000 spectrophotometer (Thermo Fisher Scientific, USA). Paired‐end sequencing (2 × 150 bp) was performed on an Illumina HiSeq platform (Illumina Inc., San Diego, CA, USA). Raw sequencing reads were subjected to quality control using FastQC to identify any issues with the data (https://www.bioinformatics.babraham.ac.uk/projects/fastqc/). Low‐quality reads and adapter sequences were trimmed using Trimmomatic with default parameters (Bolger et al. 2014). High‐quality reads were then assembled de novo using MEGAHIT (Li et al. 2016) with the following parameters: ‘–min‐contig‐len 1000 –k‐min 21 –k‐max 141 –k‐step 12 –merge‐level 20,0.95.’ Assembly quality was evaluated using QUAST to determine N50 values and contig statistics. Open reading frames (ORFs) were predicted from the assembled contigs using Prokka with default settings (Seemann 2014).

### 2.2 Metagenome Assembly, ARG Detection and Phylogenetic Analysis of Potential MBLs

Metagenome‐assembled genomes (MAGs) were reconstructed using contig binning through MetaWRAP (version 1.3.2), which integrates multiple binning algorithms and refinement steps to enhance bin quality. Taxonomic classification of the reconstructed MAGs was performed using the Genome Taxonomy Database Toolkit (GTDB‐Tk, version 2.4.0), aligning them against the Genome Taxonomy Database (GTDB) for consistent microbial taxonomy assignments (Chaumeil et al. 2019). Genome completeness and contamination were assessed using CheckM (Parks et al. 2015).

ARGs in the MAGs were detected using ABRicate (version 1.0.1) with the NCBI database as a reference (Zankari et al. 2012). Detection thresholds were set at a minimum coverage of 80% and a DNA identity of at least 80% to ensure accuracy. Insertion sequences were identified and characterised using ISfinder (https://www‐is.biotoul.fr/). Potential carbapenem resistance genes were identified using the fARGene tool (Berglund et al. 2019), while gene context analysis was conducted with EasyFig (version 2.2.5) to visualise the genomic surroundings of the ARGs. A 300‐bp fragment immediately upstream (5′ end) of the inferred ARG start codon (ATG, designated +1) was extracted from the assembled contig and analysed with BPROM (Softberry Inc.; *σ* ^70^‐promoter model, default threshold = 0.20). Predictions producing a linear discriminant function (LDF) score greater than 0.20 were retained as putative promoters; the coordinates of each predicted −35 and −10 element were recorded relative to the ARG start codon.

Potential metallo‐β‐lactamase (MBL) protein sequences derived from the MAGs, along with B1 subgroup MBL sequences obtained from the NCBI database, were aligned using the ClustalW algorithm implemented in MEGA (version 11.0.13) for multiple sequence alignment. Phylogenetic analyses were conducted using CLC Genomics Workbench 12, with evolutionary relationships inferred using the maximum likelihood method to construct phylogenetic trees. To improve the reliability of phylogenetic inferences, bootstrap analysis was performed with 1000 replicates to assess the statistical support of the branching patterns.

Following the construction of a phylogenetic tree, ancestral sequence reconstruction was performed to infer potential evolutionary intermediates along the AMM‐1 lineage. Briefly, all relevant MBL amino acid sequences were realigned using MAFFT (v7.520) with default parameters to ensure accurate multiple sequence alignment. The resulting alignment was utilised to re‐estimate phylogenetic relationships using IQ‐TREE (v2.4.0). The best‐fit amino acid substitution model was selected using ModelFinder, and branch support was evaluated by performing 1000 ultrafast bootstrap replicates.

Ancestral sequences at key internal nodes were inferred using the codeml module in the PAML package (v4.10.7) within a maximum likelihood framework, utilising the tree topology and alignment as inputs. The most likely amino acid sequence for each ancestral node was extracted from the codeml output.

To quantify evolutionary divergence along the AMM‐1 lineage, we first performed root‐to‐tip depth calculations. For each extant and ancestral taxon in the reconstructed phylogeny, cumulative branch lengths (substitutions per site) from the root to each terminal node were calculated using the IQ‐TREE‐generated tree file. These root‐to‐tip distances were extracted using custom R scripts and visualised to compare the evolutionary depth of AMM‐1 with other MBLs.

Subsequently, stepwise amino acid divergence and identity calculations were performed. Amino acid sequences of successive ancestral and descendant nodes, including AMM‐1, were compared pairwise. Absolute amino acid differences, cumulative differences and pairwise percent identity were calculated and visualised using ggplot2.

### 2.3 In Silico Structure Modelling of AMM‐1

The three‐dimensional structure of the potential MBL protein was predicted using ColabFold v1.5.5 integrated with AlphaFold2. Multiple sequence alignment was performed with the ‘UniRef + Environmental’ database to ensure comprehensive sequence coverage. The model type was set to ‘auto’, the pairing mode to ‘unpaired + paired’, and the number of recycles to 3. Five structural models were generated, each differing in their predicted Local Distance Difference Test (pLDDT) scores. The model with the highest pLDDT score was selected for further analysis.

### 2.4 Coarse‐Grained Flexibility Modelling and RMSF Analysis

The predicted AMM‐1 structure and VMB‐1, IMP‐1 and DIM‐1 crystal structures from the PDB were used for downstream analysis.

Each PDB was submitted to the CABS‐flex 3.0 web server (https://lcbio.pl/cabsflex3/) under the Flexibility mode with default settings (50 cycles, 50 frames, Cα–Cα restraints: gap = 3, min = 3.8 Å, max = 11.5 Å; temperature = 1.4; 100% restraints). Resulting fluctuation files (fluctuation.csv or rmsf.txt) were downloaded for each enzyme.

One‐letter sequences were extracted from each PDB's ATOM records and globally aligned to AMM‐1 via Biopython's pairwise2.align.globalxx. The pairwise alignment yielded residue‐to‐residue mapping dictionaries for VMB‐1, IMP‐1 and DIM‐1 onto AMM‐1 numbering.

Per‐residue Cα root‐mean‐square fluctuation (RMSF) values were loaded and processed in Python using pandas for data handling and matplotlib for plotting. Mapped RMSF profiles were overlaid to generate the final comparative flexibility plot.

### 2.5 Cloning of the AMM‐1 and AMM‐2 Genes

To assess whether the potential MBLs exhibit active β‐lactamase function, the genes encoding the proteins were cloned into the pET‐28a expression vector using a double digestion and ligation method. The AMM‐1 and AMM‐2 genes were codon‐optimised for expression in *E. coli* and synthesised by BGI Genomics Co. Ltd. (Shenzhen, China). The optimised sequences are shown in Table S1. The AMM‐1 gene was amplified by PCR using the primers AMM‐F (5′‐GGGGGATCCATGAAAGCCCTGCTGAC‐3′) and AMM‐1‐R (5′‐GGGGTCGACTTACTGATTCAGAATA‐3′), while the AMM‐2 gene was amplified using the primers AMM‐F and AMM‐2‐R (5′‐GGGGTCGACTTACTGATTCAGAATC‐3′). The PCR products were purified using the TIANgel Purification Kit (Tiangen Biotech, Beijing, China). Both the purified PCR fragments and the pET‐28a vector were digested with restriction enzymes BamHI and SalI. The digested products were then purified to remove residual enzymes and small DNA fragments. Ligation of the digested gene fragments into the linearised pET‐28a vector was conducted using T4 DNA Ligase (New England Biolabs, Beijing, China). The recombinant plasmid, designated as AMM‐1‐pET‐28a and AMM‐2‐pET‐28a, was transformed into *E. coli* BL21(DE3) competent cells (TransGen Biotech, Beijing, China) via heat shock transformation, respectively. An empty pET‐28a vector without the gene insert was also transformed into BL21(DE3) cells to serve as a negative control.

### 2.6 Carba NP Test

We then used a previously reported method, ‘CNPt‐direct’ (Pasteran et al. 2015), for direct carbapenemase detection from bacterial cultures. Briefly, BL21(DE3) cells carrying the pET‐28a plasmid were grown overnight at 37°C on Mueller‐Hinton agar (MHA) (OXOID, Hampshire, UK), scraped off with a 1‐μL loop and directly suspended in 100 μL of CNPt‐direct mix containing 0.1% v/v Triton X‐100, 0.05% phenol red, 0.1 mmol/L ZnSO_4_ and 6 or 0 mg/mL imipenem. After vigorous mixing for 5–10 s, the suspension was incubated at 35°C for 30 min. Colour change from red to yellow indicated imipenem degradation by the MBLs.

### 2.7 Antimicrobial Susceptibility Testing

According to CLSI guidelines, the minimum inhibitory concentration (MIC) of *E. coli* was determined using the broth dilution method. Briefly, colonies from freshly cultured BL21(DE3) plates were inoculated into PBS, and the turbidity was adjusted to 1 × 10^6^ CFU/mL (1/100th of the 0.5 McFarland standard). Then, two‐fold serial dilutions of each antibiotic solution were prepared in Mueller‐Hinton Broth (MHB) (OXOID, Hampshire, UK), covering the expected MIC range. Each concentration of the antibiotic solution was dispensed into sterile 96‐well microtiter plates, with 100 μL per well. Then, 100 μL of the bacterial suspension was added to each well. The plates were incubated at 37°C in the dark for 16 h, and turbidity in each well was observed. In the MIC assays, NDM‐1 was included as a parallel control to enable a more direct comparison of the resistance phenotype conferred by AMM‐1. All MIC experiments were repeated three times.

### 2.8 Nucleotide Sequence Accession Number

The MAG generated in this study is available in the NCBI database (accession number: RJNA1211733).

## 3 Results

### 3.1 Identification of the Novel MBL AMM‐1

**FIGURE 1:**
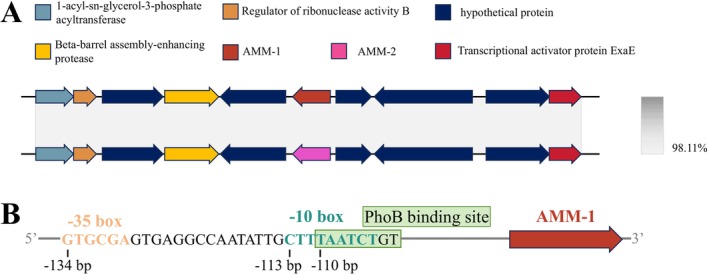
Genomic context of the AMM‐1 and AMM‐2 genes in *Alteromonas mangrovi*. (A) The diagram shows the genetic arrangement of genes flanking AMM‐1 and AMM‐2 in the *A. mangrovi* chromosome. The AMM‐1 and AMM‐2 genes are depicted in red and pink, respectively, while the surrounding genes are represented by different coloured boxes indicating their respective functions. Functional colour annotations are shown above; arrow direction indicates ORF orientation. Sequence identity (98.11%) between the AMM‐1 and AMM‐2 regions, including their flanking genes, is shown on the right. (B) Predicted promoter region upstream of AMM‐1. The −35 box (GTGCGA at −134 bp), −10 box (CTTTAATCT at −113 bp) and PhoB binding site (TAATCTGT at −110 bp) were identified by BPROM. All positions are relative to the first nucleotide of the AMM‐1 start codon (+1).

Using FarGene analysis of metagenome‐assembled genomes (MAGs), a novel metallo‐β‐lactamase (MBL) gene, designated AMM‐1, was identified within the MAG PMC‐AMM‐1, which was derived from a water sample collected at the Yangtze River estuary. The AMM‐1 gene is 732 bp in length and encodes a protein consisting of 243 amino acids. It is located on a 26,585 bp contig with a GC content of 50.96%. Taxonomic classification using GTDB‐Tk assigned the MAG to *A. mangrovi* , which exhibited a genome completeness of 93.23% and a contamination rate of 0.89%. To evaluate the homology of AMM‐1, we conducted a BLASTp search against the NCBI database, identifying a closely related protein (WP_140959230.1) on the chromosome of *A. mangrovi* strain GS‐14, which we designated as AMM‐2 (Figure 1A). This strain was isolated from mangrove wetland sediments in the Beilun Estuary National Nature Reserve, Guangxi, China. AMM‐2 shares 99% coverage and 97.12% nucleotide sequence identity with AMM‐1. Both AMM‐1 and AMM‐2 are positioned within conserved chromosomal regions of *A. mangrovi* , adjacent to essential housekeeping genes. BPROM analysis of the 300 bp region immediately upstream of AMM‐1 predicted a *σ* ^70^‐type promoter (LDF = 1.85) with a canonical −35 box (GTGCGA) located 134 bp upstream of the AMM‐1 start codon and a −10 box (CTTTAATCT) situated 113 bp upstream; a PhoB binding site (TAATCTGT) overlaps this region at −110 bp, indicating that endogenous transcription in *A. mangrovi* may be induced under phosphate‐limiting or related environmental conditions (Figure 1B).

Although no immediate mobile genetic elements (MGEs) were found adjacent to AMM‐1, the possibility of future mobilisation cannot be ruled out under selective pressures, which may facilitate horizontal gene transfer into clinically relevant pathogens. This genomic arrangement suggests a limited potential for horizontal gene transfer, indicating that AMM‐1 and AMM‐2 are likely maintained through vertical inheritance within *A. mangrovi* populations.

### 3.2 Phylogenetic Classification of AMM‐1

**FIGURE 2:**
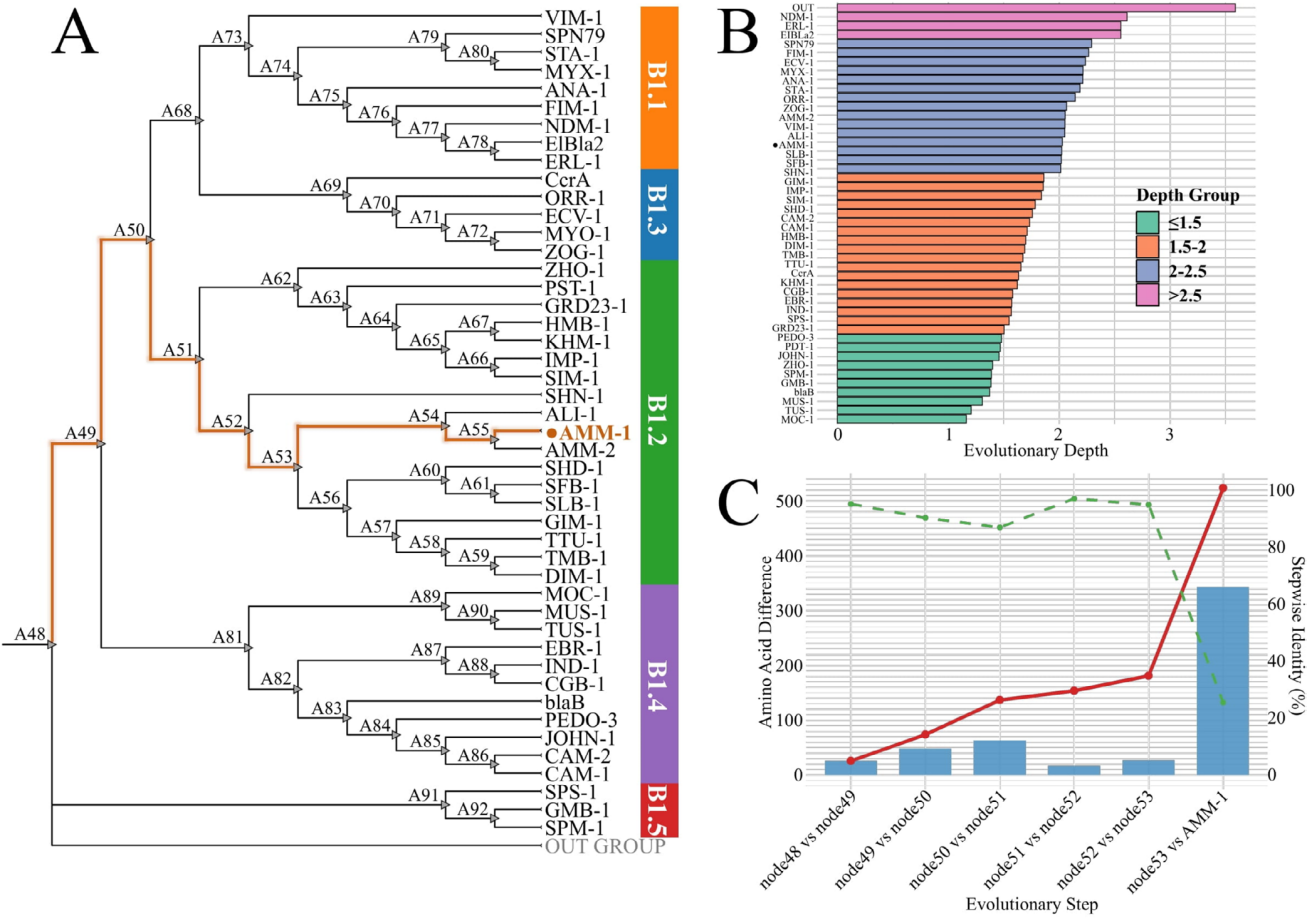
Phylogenetic and evolutionary analysis of AMM‐1. (A) A maximum likelihood tree was constructed using the amino acid sequence of the novel MBLs AMM‐1 (highlighted in orange with a solid dot) alongside representative B1 class MBLs, including IMP‐1, VIM‐2 and others retrieved from the NCBI database. The tree was inferred using the Jones‐Taylor‐Thornton (JTT) model and bootstrap analysis with 1000 replicates. Internal node labels (A48–A92) correspond to ancestral sequences reconstructed via PAML. The orange branch trace denotes the inferred evolutionary trajectory from deep ancestral nodes to AMM‐1. Right‐side coloured bars denote intra‐subclass divisions within B1 (B1.1–B1.5). (B) Root‐to‐tip evolutionary depth of each MBL sequence derived from the phylogeny in (A), calculated as the cumulative branch length from the root to each extant taxon. Bars are colour‐coded by depth range: ≤ 1.5 (green), 1.5–2 (orange), 2–2.5 (blue) and > 2.5 (purple), as indicated in the legend. AMM‐1 is highlighted with a solid dot; ‘OUT’ denotes the outgroup. (C) AMM‐1 evolutionary divergence gradient along the inferred path from node48 to AMM‐1. The bar chart (left *y*‐axis) shows amino acid changes at each evolutionary step. The red line tracks cumulative amino acid differences (left *y*‐axis), and the green dashed line indicates pairwise identity between each parent and descendant node (right *y*‐axis).

To elucidate the evolutionary relationships of the novel AMM‐1 gene, we performed a maximum‐likelihood phylogenetic analysis. The resulting tree (Figure 2A) demonstrates that AMM‐1 closely clusters with established B1.2 MBLs, including IMP‐1 and VMB‐1. This close association confirms the classification of AMM‐1 within the B1.2 subclass, suggesting that it possesses similar enzymatic properties and contributes to carbapenem resistance.

Based on the maximum‐likelihood phylogeny, we computed root‐to‐tip branch lengths (substitutions/site) for each terminal taxon. Depth values ranged from 1.16 to 3.59, with an average of 1.85. AMM‐1 exhibited a depth of 2.02, ranking 17th out of 47 (upper third) among surveyed MBLs (Figure 2B). Its closest relatives, AMM‐2 and ALI‐1, had depths of 1.78 and 1.60, respectively. Several well‐characterised MBLs clustered at various depths: NDM‐1 (2.61), VIM‐1 (2.05), IMP‐1 (1.84) and SPM‐1 (1.39) (Figure 2B, Table S2). Therefore, AMM‐1 is evolutionarily deeper than IMP‐1 and SPM‐1, similar in depth to VIM‐1, but markedly shallower than the clinically significant NDM‐1.

Ancestral sequence reconstruction using PAML identified the evolutionary pathway: node48 → node49 → node50 → node51 → node52 → node53 → AMM‐1 (Table S3). Pairwise comparisons between consecutive nodes (Figure 2C; Table S4) revealed moderate incremental differences for the first five transitions, ranging from 17 to 63 substitutions with identities of 86%–96%. In the final transition from node53 to AMM‐1, 343 substitutions were inferred (25% identity), substantially increasing cumulative differences from 181 to 524 residues over the entire 524‐amino‐acid alignment. Overall, AMM‐1 accumulated 524 amino‐acid substitutions relative to node48, with the final transition contributing the majority of these changes.

We further compared AMM‐1 to six previously reported environmental MBLs (Table 1). These enzymes showed considerable ecological diversity, originating from marine aquaculture pathogens (VMB‐1), marine fish pathogens (ALI‐1), psychrophilic Antarctic marine bacteria (SLB‐1, SFB‐1), marine alphaproteobacteria (ElBla2) and uncultured estuarine bacteria (CAM‐2). Regarding genetic mobility, only VMB‐1 is plasmid‐borne, carried by an integron‐containing conjugative IncC plasmid. The other analysed MBLs (ALI‐1, SLB‐1, SFB‐1, ElBla2 and CAM‐2) are chromosomally encoded without known adjacent mobile elements, indicating limited horizontal transfer potential. Amino acid identity between AMM‐1 and these MBLs varied significantly, ranging from 10.4% (ALI‐1) to 36.6% (VMB‐1). Collectively, the observed variability in ecological origin, genetic context and sequence conservation underscores the substantial heterogeneity among environmental MBLs, emphasising AMM‐1's distinct evolutionary trajectory and potential dissemination dynamics.

**TABLE 1:** Representative environmental MBLs and their identity to AMM‐1.

MBL	Source host(s)	Genetic mobility	Identity	References
VMB‐1	*Vibrio alginolyticus strain* Vb1796 (food‐borne marine isolate)	Plasmid‐borne: integron‐carried on conjugative IncC plasmid pVB1796	36.6%	Zheng et al. (2020)
ALI‐1	*Aliivibrio salmonicida* (marine fish pathogen; also found in other Aliivibrio spp. in nature)	Chromosomal (intrinsic, not on mobile elements)	10.4%	Kristiansen et al. (2015)
SLB‐1	*Shewanella livingstonensis* (psychrophilic marine bacterium from Antarctic waters)	Chromosomal (intrinsic; environmental reservoir, no known mobility)	33.6%	Kang et al. (2023)
SFB‐1	*Shewanella frigidimarina* (psychrophilic marine bacterium from Antarctic waters)	Chromosomal (intrinsic; environmental reservoir, not on known mobile elements)	34.9%	Poirel et al. (2005)
ElBla2	*Erythrobacter litoralis* HTCC2594 (marine Alphaproteobacterium)	Chromosomal (no nearby mobile genetic elements)	28.4%	Jiang et al. (2018)
CAM‐2	Uncultured bacterium (MAG in family Pyrinomonadaceae, estuary environment); also detected in *Pseudomonas* from hospital sewage	Chromosomal (identified on MAG contig; no integron or transposon in vicinity reported)	24.8%	Xiang and Li (2022)

### 3.3 Identification of Di‐Zinc Catalytic Center of AMM‐1

**FIGURE 3:**
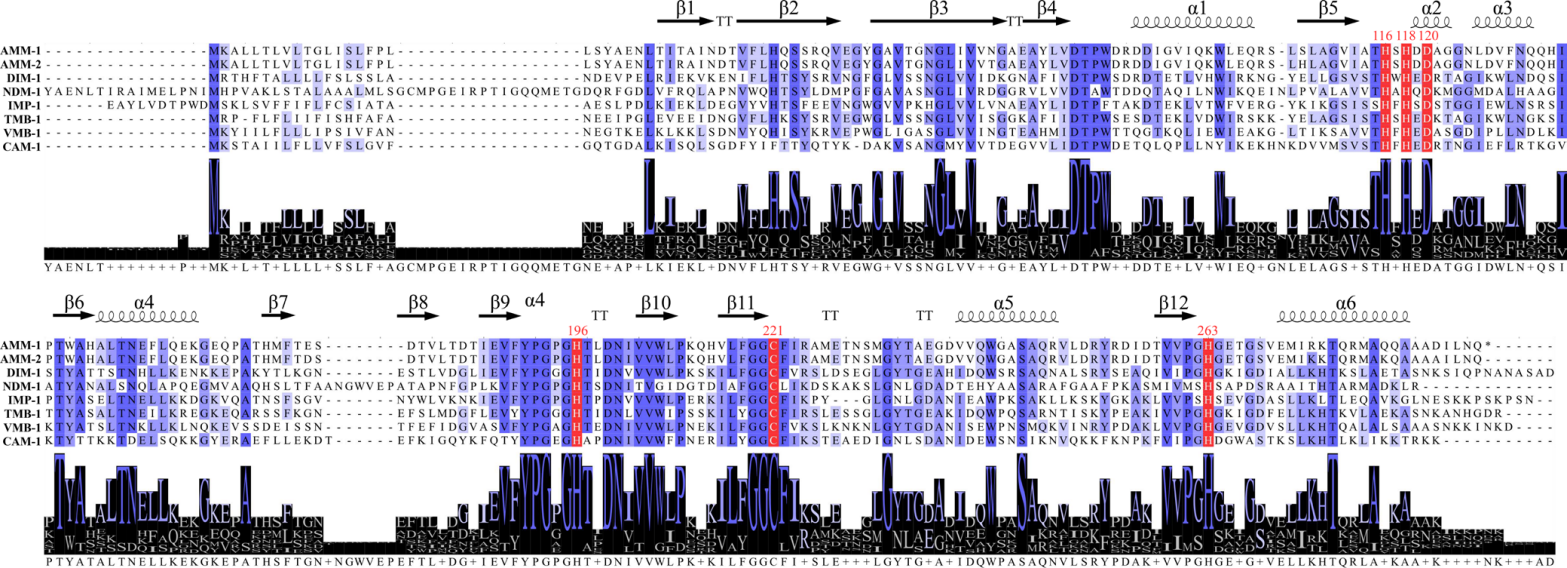
AQ3Sequence alignment of the di‐zinc catalytic center in AMM‐1, AMM‐2 and representative B1 MBLs. Multiple sequence alignment highlighting the conserved residues responsible for zinc ion coordination in the di‐zinc catalytic center. Amino acid residues are shown using single‐letter abbreviations. The key amino acids interacting with Zn1 and Zn2 are highlighted in red and numbered based on the standard MBL numbering system. Conserved secondary structure elements are indicated above the alignment: α denotes α‐helices, β denotes β‐strands and TT represents turn or loop regions connecting secondary structure elements.

Zinc ions in MBLs are crucial for attacking the β‐lactam ring, which leads to antibiotic degradation. A di‐zinc‐dependent catalytic centre is a hallmark of B1 MBLs (King and Strynadka 2011; Ortega‐Balleza et al. 2024). To characterise the catalytic centre of AMM‐1, we performed a detailed alignment of the AMM‐1 amino acid sequence with representative MBLs (Figure 3). The alignment revealed that AMM‐1 maintains complete conservation of key histidine (His) and aspartate (Asp) residues essential for zinc ion coordination, specifically His116, His118, Asp120 and His196. These residues facilitate the binding of two zinc ions (Zn1 and Zn2) within the active site, mirroring the coordination environment observed in established B1 MBLs (Yamamoto et al. 2022). This conserved coordination pattern ensures the structural integrity and catalytic functionality of AMM‐1, enabling the hydrolysis of the β‐lactam ring in carbapenem antibiotics.

**FIGURE 4:**
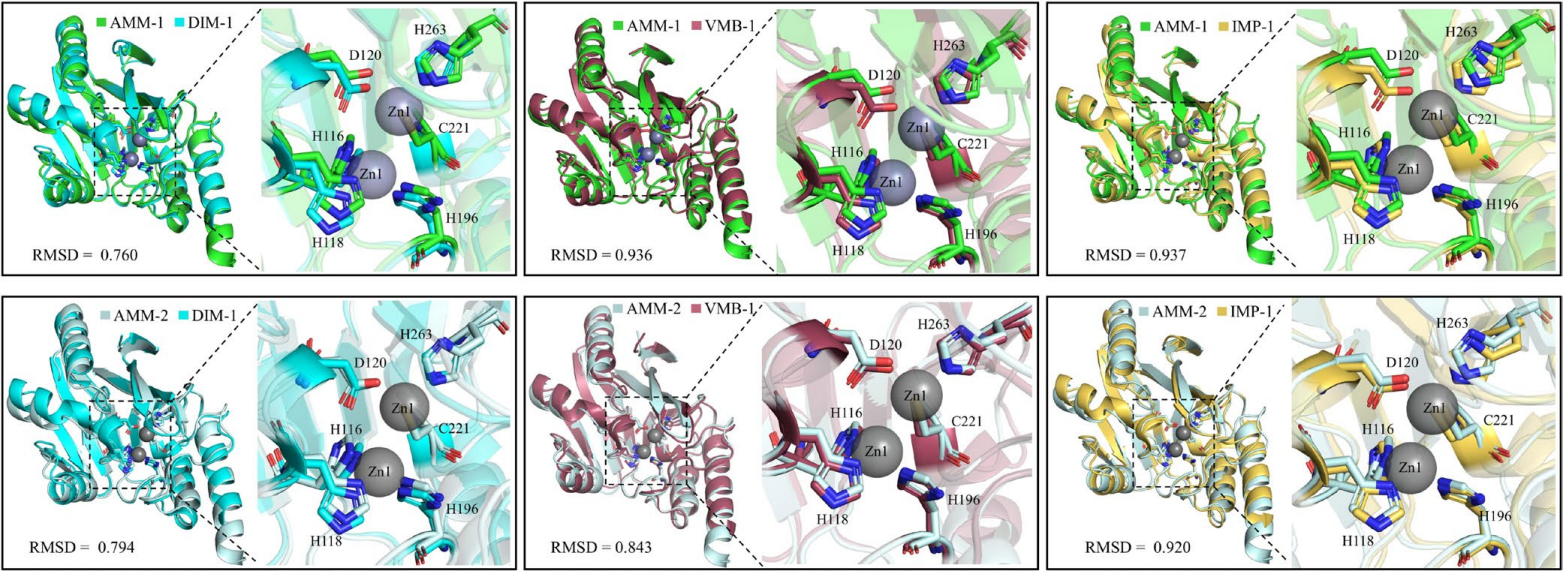
Structural comparison of AMM‐1, AMM‐2 and representative MBLs. The root‐mean‐square deviation (RMSD) values are annotated beneath each alignment. Zinc ions (Zn) are represented as grey spheres. The active site structure is prominently magnified to showcase the six residues that interact with Zinc (Zn), depicted in a stick representation to highlight their spatial arrangement and atomic details. All Zn‐interacting residues are labelled using the one‐letter amino acid code. Structural comparison of AMM‐1, AMM‐2 and representative MBLs. The root‐mean‐square deviation (RMSD) values are annotated beneath each alignment. Zinc ions (Zn) are represented as grey spheres. The active site structure is prominently magnified to showcase the six residues that interact with Zinc (Zn), depicted in a stick representation to highlight their spatial arrangement and atomic details. All Zn‐interacting residues are labelled using the one‐letter amino acid code.

To further confirm the enzymatic characteristics of AMM‐1 and AMM‐2 as typical B1 MBLs, we predicted their 3D structures using AlphaFold 2 and compared them with well‐characterised B1 MBLs including DIM‐1, VMB‐1 and IMP‐1 (PDB accession numbers: 4wd6, 6jv4 and 7xhw) (Figure 4). The structural alignment focused on identifying active sites, metal ion‐binding sites and potential interactions with substrates or inhibitors, thereby elucidating the enzymatic function and resistance mechanisms of AMM‐1.

The root mean square deviation (RMSD) values between AMM‐1 and its counterparts DIM‐1, VMB‐1 and IMP‐1 are 0.760 Å, 0.936 Å and 0.937 Å, respectively, while the RMSD values for AMM‐2 in comparison with DIM‐1, VMB‐1 and IMP‐1 are 0.794, 0.843 and 0.920 Å, respectively. These low RMSD values indicate a high degree of structural similarity between AMM‐1 and AMM‐2 with DIM‐1, VMB‐1 and IMP‐1. Both proteins display a conserved MBL fold essential for the hydrolysis of β‐lactam antibiotics. Notably, six zinc‐binding residues in AMM‐1 and AMM‐2 align closely with those in representative MBLs, exhibiting similar spatial arrangements that facilitate zinc ion coordination. Zn1 is coordinated by His116, His118 and His196 in a tetrahedral geometry, while Zn2 is coordinated by Asp120, Cys221 and His263, also forming a tetrahedral configuration (Yamamoto et al. 2022). This precise geometric configuration of the two zinc ions is critical for the catalytic efficiency of MBLs in cleaving the β‐lactam ring. The structural resemblance between AMM‐1, AMM‐2 and the representative MBLs supports the hypothesis that AMM‐1 and AMM‐2 operate through a zinc‐dependent mechanism, thereby contributing to antibiotic resistance by effectively hydrolysing β‐lactam antibiotics. This structural analysis not only reinforces the classification of AMM‐1 and AMM‐2 as B1 metallo‐β‐lactamases but also provides deeper insights into their molecular basis for enzymatic activity and resistance functionality.

### 3.4 Enhanced Backbone Flexibility of AMM‐1 Compared to VMB‐1, IMP‐1 and DIM‐1

**FIGURE 5:**
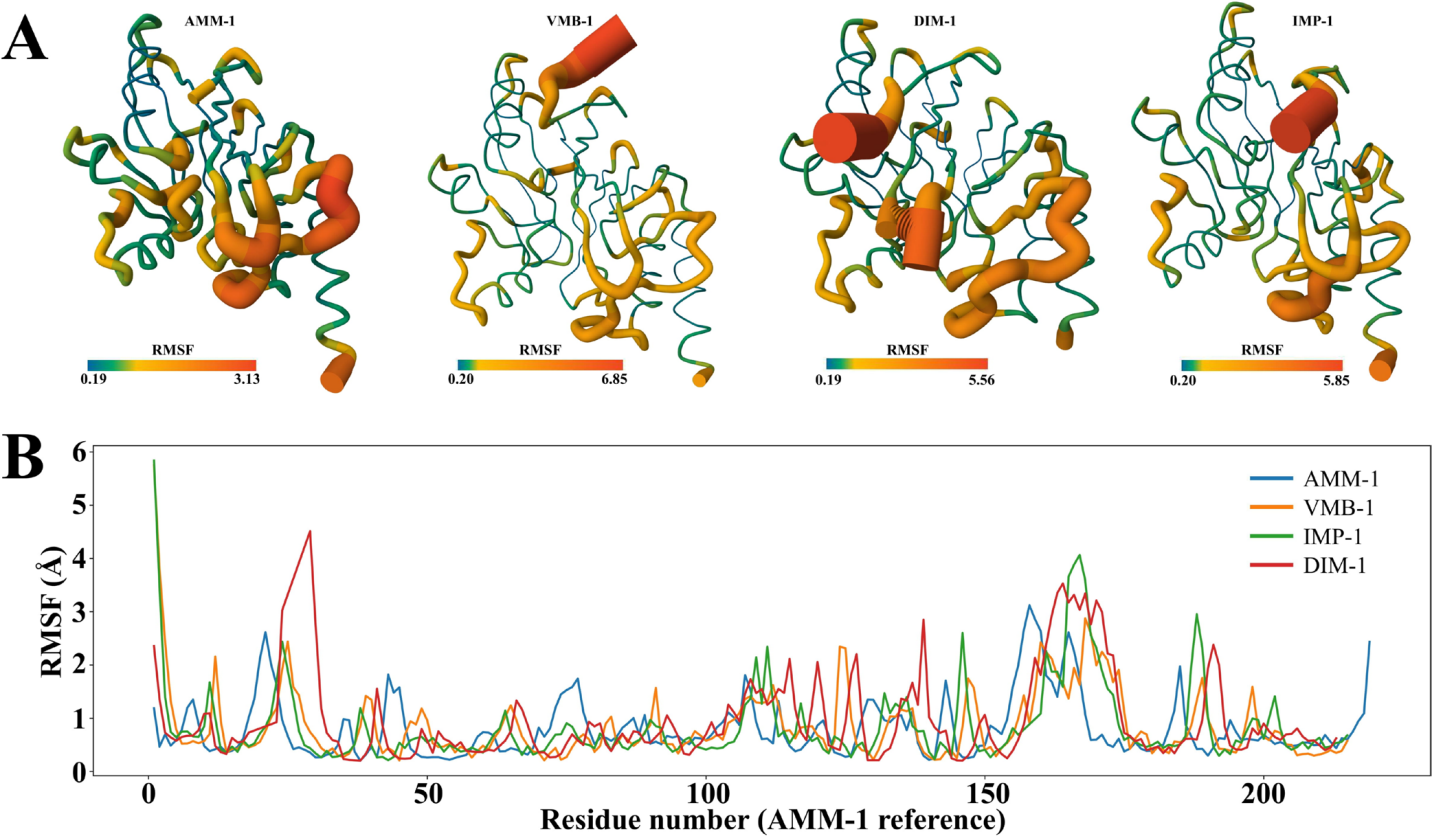
Comparative flexibility analysis of AMM‐1, VMB‐1, IMP‐1 and DIM‐1. (A) Three‐dimensional ribbon representations of the predicted flexibility profiles for AMM‐1, VMB‐1, IMP‐1 and DIM‐1. Structures are colour‐coded by residue‐level root‐mean‐square fluctuation (RMSF) values, with the spectrum ranging from blue (low flexibility) to red (high flexibility). The thickness of each ribbon corresponds to the magnitude of local flexibility. (B) RMSF (Å) plots quantifying residue‐level fluctuations across the four MBLs, aligned according to AMM‐1 residue numbering. AMM‐1 (blue), VMB‐1 (orange), IMP‐1 (green) and DIM‐1 (red) exhibit distinct flexibility signatures, particularly in regions corresponding to surface loops and the active‐site vicinity.

The flexibility modes of AMM‐1, VMB‐1, IMP‐1 and DIM‐1 were simulated and compared. The resulting overlay revealed that AMM‐1 exhibits a distinct dynamic profile compared to the other MBLs, particularly in the active‐site loops and adjacent secondary structural elements (Figure 5A). In loop L3, a flexible segment located between β2 and β3 strands flanking one side of the active‐site groove in subclass B1 MBLs, all enzymes exhibited pronounced RMSF peaks, confirming its role as a major flexible element (Figure 5B). AMM‐1's L3 loop was noticeably less flexible compared to that of other MBLs. The maximum RMSF value for AMM‐1 in this region reached approximately 2.6 Å, occurring slightly N‐terminal to the aligned positions of peaks in other enzymes. In contrast, DIM‐1 exhibited the highest fluctuations in the L3 loop region. IMP‐1 and VMB‐1 showed intermediate flexibility in their L3 loops, with RMSF values around 2.4 Å. Therefore, the reduced flexibility of AMM‐1's L3 loop relative to DIM‐1 may indicate a more restricted yet potentially more stable substrate binding mode. Conversely, DIM‐1's highly mobile L3 could confer greater adaptability—though possibly at the cost of stability—in accommodating diverse β‐lactams.

Loop L10, a longer active‐site loop of approximately 20 residues, is situated on the opposite side of the substrate pocket and typically covers the metal‐binding cleft. Similar to L3, loop L10 is recognised as a flexibility hotspot in B1 MBLs (Piccirilli et al. 2021). All four enzymes demonstrated significant RMSF peaks within the L10 region (residues 150–170, Figure 5B). However, both the pattern and magnitude of L10 flexibility in AMM‐1 differed markedly from those of the other enzymes. AMM‐1 was most flexible in residues 155–160, reaching an RMSF maximum of approximately 2.9 Å; however, the remainder of its L10 loop (residues 160–170) was more rigid compared to the other enzymes. For instance, the central L10 region of IMP‐1 and DIM‐1 fluctuated between approximately 3 and 4 Å, whereas the RMSF for AMM‐1 decreased below 2 Å in the corresponding aligned segment. Collectively, these findings reveal that AMM‐1 possesses unique structural dynamics in functionally critical regions, distinguishing it clearly from DIM‐1, IMP‐1 and VMB‐1.

### 3.5 Antibiotic Resistance Phenotype Conferred by AMM‐1 and AMM‐2

To characterise the antibiotic resistance phenotype conferred by the novel AMM‐1 and AMM‐2 MBLs, we cloned the genes into a susceptible *E. coli* strain and performed MIC assays against a panel of β‐lactam antibiotics, including carbapenems. The *E. coli* strain expressing the novel MBLs demonstrated a significant increase in resistance to carbapenem antibiotics compared to the control strain harbouring an empty vector. MIC assays revealed a significant increase in resistance to carbapenem antibiotics in *E. coli* strains expressing AMM‐1 and AMM‐2, with MIC values for imipenem, ertapenem and meropenem rising from < 0.25 μg/mL in the control strain to 4, 32 and 64 μg/mL, respectively (Table 2). Additionally, resistance to other β‐lactams, such as ceftazidime, cefotaxime and cefazolin, was markedly enhanced, with MICs increasing by approximately 256‐fold, 256‐fold and 128‐fold, respectively. In contrast, no significant changes in MIC values were observed for non‐β‐lactam antibiotics, indicating that the resistance phenotype is specific to β‐lactam compounds. Notably, all tested strains remained sensitive to aztreonam, consistent with previous reports that MBLs, including NDM‐1 positive control, generally do not hydrolyse this monobactam antibiotic due to its unique chemical structure (Walsh et al. 2005; Nordmann et al. 2021).

**TABLE 2:** MIC profiles of AMM‐1 and AMM‐2 expressing *E. coli* strains. MIC assays were conducted to evaluate the resistance conferred by AMM‐1 against various antibiotics.

Antibiotic	MIC (μg/mL)^a^				CLSI R breakpoint (μg/mL)
	BL21/pET28a‐AMM‐1	BL21/pET28a‐AMM‐2	BL21/pET28a‐NDM‐1	BL21/pET28a (control)	
Ampicillin	> 128	> 128	> 128	1	32
Amoxicillin	> 128	> 128	> 128	0.5	32
Piperacillin	> 128	> 128	> 128	< 0.25	64
Ceftriaxone	> 128	> 128	> 128	< 0.25	4
Ceftazidime	64	64	> 128	0.25	16
Cefepime	0.5	0.5	64	< 0.25	8
Cefotaxime	64	64	> 128	< 0.25	4
Cefazolin	64	64	> 128	0.5	8
Aztreonam	< 0.25	< 0.25	< 0.25	< 0.25	16
Meropenem	64	64	32	< 0.25	4
Imipenem	4	4	64	< 0.25	4
Ertapenem	32	32	32	< 0.25	2
Gentamicin	0.25	0.25	0.25	0.25	16
Tobramycin	0.5	0.5	0.5	0.25	16
Ciprofloxacin	< 0.25	< 0.25	< 0.25	< 0.25	1
Doxycycline	0.5	0.5	0.5	0.5	4
Chloramphenicol	0.25	0.25	0.25	0.25	32
Tetracycline	2	2	2	2	4

^a^ MICs reproduced in triplicate with identical results. No variation observed.

To further validate the carbapenemase activity of AMM‐1 and AMM‐2, Carba NP tests were performed, yielding positive results that corroborate the MIC assay findings (Figure S1). Collectively, these results demonstrate that AMM‐1 effectively hydrolyses β‐lactam antibiotics, particularly carbapenems, thereby conferring high‐level resistance and highlighting its potential role in the dissemination of antimicrobial resistance.

## 4 Discussion

The identification and characterisation of the novel B1.2 MBL AMM‐1 from an *A. mangrovi* MAG highlights the importance of environmental reservoirs in the global antibiotic resistance landscape. Environmental gene pools frequently seed resistance determinants that later surface in hospitals, a point underscored by recent global analyses (Brüssow 2024). Moreover, chromosomally located carbapenemase genes have been shown to excise and disseminate across borders (Abe et al. 2023; Wang et al. 2024), placing discoveries like AMM‐1 within an international One‐Health context. Our findings show that AMM‐1 shares key structural and functional features with clinically significant B1 MBLs, such as IMP‐1 and VMB‐1, including the conserved di‐zinc‐dependent catalytic mechanism essential for β‐lactam hydrolysis (Figure 4). Beyond confirming that AMM‐1 can confer carbapenem resistance in a susceptible *E. coli* host, these results underscore the notion that clinically challenging resistance genes may emerge and persist in natural habitats long before their potential mobilisation into clinically relevant pathogens.

It is increasingly recognised that many of the ARGs prevalent in Enterobacteriaceae originated from environmental bacteria (Mills and Lee 2019; Wu et al. 2019; Zhang, Dong, et al. 2020; Zhang, Fukuda, et al. 2020; Berglund et al. 2021). These environmental antecedents may have evolved their β‐lactamase activities in response to naturally occurring β‐lactam compounds produced by environmental microorganisms or through long‐standing ecological competition. Over time, anthropogenic pressures—such as the discharge of untreated wastewater, agricultural runoff containing veterinary antibiotics and heavy metal pollution—can select and enrich these resistance determinants, raising the likelihood of their horizontal transfer (Zhang, Dong, et al. 2020; Zhang, Fukuda, et al. 2020; Bottery et al. 2021; Liu et al. 2021). Although our study does not include direct chemical measurements from the sampling site, previous environmental monitoring surveys in the Yangtze River Estuary have consistently detected multiple classes of anthropogenic pollutants. These include antibiotics such as tetracyclines, sulfonamides and fluoroquinolones, frequently reported in surface sediments and water columns (Yan et al. 2013; Shi et al. 2014), as well as heavy metals like cadmium (Cd) and mercury (Hg), which have been observed at ecologically significant concentrations (Liu et al. 2024). These environmental stressors are widely recognised for their ability to exert selective pressure on microbial communities, promoting the persistence and potential enrichment of resistance genes in situ. In the context of our findings, such regional contamination provides a plausible ecological backdrop that may contribute to the maintenance of AMM‐1 in its environmental host.

Although no integrons, transposons or plasmids were identified in the immediate genomic vicinity of AMM‐1, suggesting the gene is not currently mobilisable, its presence in an environmental bacterium underscores a potential long‐term risk. Environmental microbial communities are inherently dynamic, and under selective pressures such as antibiotic exposure, horizontal gene transfer mechanisms—including the acquisition of mobile elements—can facilitate the mobilisation of chromosomal resistance genes (Berendonk et al. 2015; Zheng et al. 2021). While there is no current evidence that AMM‐1 is mobile, we highlight this as a potential threat warranting future surveillance, particularly given the phylogenetic distance between Alteromonas and clinically relevant taxa such as Enterobacterales. Recent work underscores that chromosomally encoded β‐lactamases do not represent an evolutionary ‘dead end’. For instance, Abe et al. (2023) demonstrated that meropenem stress can excise a chromosome‐integrated IMP‐6 plasmid module and disseminate it via conjugation. Experimental evolution by Zongo et al. (2024) further revealed an antiplasmid system (ApsAB) that drives stable chromosomal integration of bla_OXA‐48_ yet still allows subsequent mobilisation. Population‐based surveillance in Israel shows that > 70% of *E. coli* carrying OXA‐48–like carbapenemases now harbour these genes on the chromosome, with multiple clonal and plasmid‐mediated outbreaks recorded between 2016 and 2023 (Temkin, et al. 2025). Together, these findings indicate that AMM‐1, although currently chromosomal, may follow a similar ‘mobilise–integrate–remobilize’ cycle via integrative and conjugative elements or lateral transduction, emphasising the public health importance of monitoring chromosomal reservoirs of carbapenemase genes.

While *A. mangrovi* is phylogenetically distinct from Enterobacteriaceae, belonging to separate orders within the Gammaproteobacteria, evolutionary history has demonstrated that MBLs and other β‐lactamases can transcend substantial taxonomic boundaries through horizontal gene transfer (Berglund et al. 2021). The persistence of AMM‐1 within a stable chromosomal context may reflect a long‐standing evolutionary relationship, where the MBL gene is maintained for adaptive benefit in niche‐specific competition. Changes in environmental conditions—such as shifts in salinity, nutrient availability or increased antibiotic concentrations due to human activity—could facilitate the recruitment of MGEs that capture and mobilise the AMM‐1 gene. Once mobilised, the gene could potentially enter opportunistic pathogens in estuarine or coastal waters, eventually finding its way into human‐associated Enterobacteriaceae through human‐environment interfaces (Zheng et al. 2021).

From a public health perspective, the presence of a potent carbapenemase gene in an environmental organism like *A. mangrovi* —a bacterium not typically associated with human infection—highlights the importance of environmental surveillance, even in the absence of current evidence for gene mobility. Environmental monitoring has revealed that estuaries and other aquatic systems act as genetic ‘trading grounds’ where strains with diverse resistance elements coexist, exchange genetic material and adapt to anthropogenic pressures (Zhu et al. 2017; Ding et al. 2024). Early detection of such resistance determinants, coupled with surveillance of resistance gene flow from environmental to clinical settings, is critical to anticipating and mitigating future resistance problems. The identification of AMM‐1 and its structural and functional similarity to clinically relevant MBLs offers insight into the evolutionary potential of environmental resistance genes, although its current lack of mobilizability suggests a limited immediate clinical risk.

In addition, this work highlights the need for integrated strategies encompassing environmental microbiology, clinical epidemiology and molecular genetics to trace the origin and fate of emerging resistance determinants. Efforts to mitigate antibiotic release into ecosystems, combined with improved wastewater treatment and controlled use of antimicrobials in agriculture and aquaculture, may help reduce the environmental selective pressures that foster these resistance reservoirs (Zhu et al. 2017). Ultimately, a better understanding of the evolutionary and ecological context in which these genes arise and evolve can inform policies and interventions aimed at preserving the effectiveness of current antibiotic therapies.

## 5 Conclusion

In summary, the discovery of the AMM‐1 MBL gene within an *A. mangrovi* MAG from the Yangtze River Estuary reveals a potent carbapenem resistance determinant embedded in an environmental bacterial lineage. Although AMM‐1 is currently chromosomally encoded and lacks adjacent mobile genetic elements, its structural and phylogenetic relatedness to clinically relevant B1 MBLs raises public health concerns, as such genes may, under certain ecological or selective conditions, be horizontally transferred via mobile genetic elements. This study reinforces the critical role of environmental reservoirs as silent incubators of resistance genes, highlights the evolutionary and ecological complexity underpinning antibiotic resistance and underscores the pressing need for robust surveillance and environmental stewardship to prevent future clinical dissemination.

## Author Contributions

**Xuan Wu:** conceptualization, methodology, software, formal analysis, writing – original draft, funding acquisition. **Xinjing Han:** methodology, software, formal analysis. **Lu Zhu:** methodology, funding acquisition. **Ningning Pi:** methodology, investigation, funding acquisition. **Yi Li:** conceptualization, methodology, validation, formal analysis, writing – original draft. **Rong Xiang:** conceptualization, validation, writing – review and editing, resources.

## Conflicts of Interest

The authors declare no conflicts of interest.

## Supporting information


Figure S1.

**Table S1.** Codon‐Optimised AMM‐1 and AMM‐2 Nucleotide Sequences.

**Table S2.** Root‐to‐Tip Evolutionary Depths of AMM‐1 and Reference MBLs.

**Table S3.** Ancestral Amino Acid Sequences Inferred by Maximum Likelihood.

**Table S4.** Evolutionary Divergence Gradient from Ancestral Nodes to AMM‐1.

## Data Availability

The data that support the findings of this study are available on request from the corresponding authors. The data are not publicly available due to privacy or ethical restrictions.
