# Supplementary material for: A Novel Metallo‐β‐Lactamase AMM‐1 From Alteromonas mangrovi Reveals a Cryptic Environmental Reservoir of Carbapenem Resistance
Source: Microb Biotechnol. 2025 Jul 8;18(7):e70191. doi: 10.1111/1751-7915.70191 (PMC12238668; doi:10.1111/1751-7915.70191)

**Figure S1. Results of the Carba NP test.** The first row shows the reaction containing the antibiotic (imipenem); the second row represents the negative control without the antibiotic.


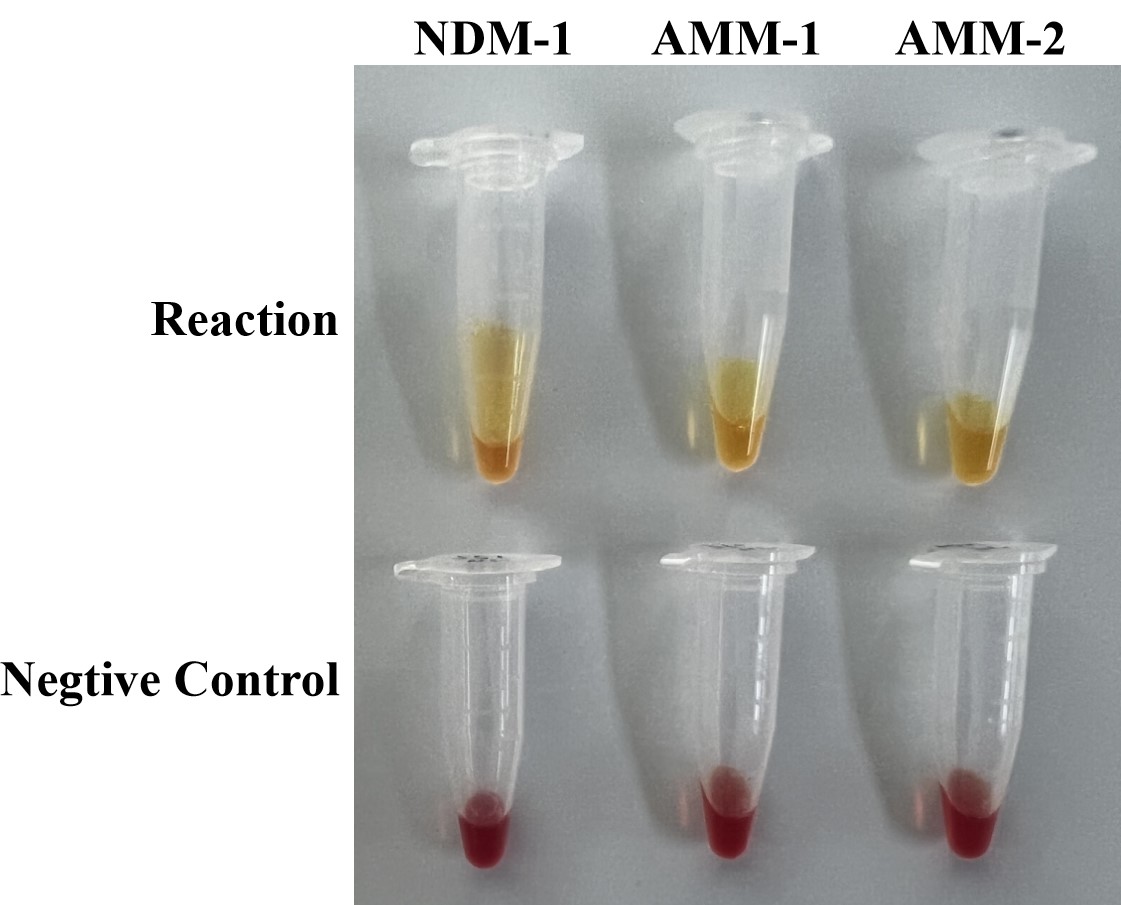

Supplement: Supplementary file 1 — Figure S1. [file MBT2-18-e70191-s002.docx]
